# Frequent residential mobility among American Indians and early indications of sexual risk among young adolescents

**DOI:** 10.1371/journal.pone.0218445

**Published:** 2019-06-17

**Authors:** Nicole R. Tuitt, Nancy Rumbaugh Whitesell, Nancy L. Asdigian, Carol E. Kaufman

**Affiliations:** 1 School of Public Health, University of California Berkeley, Berkeley, California, United States of America; 2 Prevention Research Center—Pacific Institute for Research and Evaluation, Berkeley, California, United States of America; 3 Centers for American Indian and Alaska Native Health, Colorado School of Public Health, University of Colorado Anschutz Medical Campus, Aurora, Colorado, United States of America; Universidad de Tarapaca, CHILE

## Abstract

American Indian and Alaska Native (AI/AN) youth are more likely to ever have had sex, and to have engaged in sexual activity prior to age 13 compared to all other race groups. It is essential to understand the development of skills to refuse sexual experience in early adolescence in order to reduce disparities associated with early sexual debut among AI/AN youth. Familial, social, and individual factors can act as protective influences on adolescent sexual experience; however, in other settings, research has shown that frequent residential mobility disrupts these protective influences and may increase the likelihood of adolescent sexual activity. AI/AN youth are highly mobile, and, as a result, may be especially vulnerable to increased sexual risk. To date, no prior study has considered the impact of residential mobility on AI/AN youth sexual experience, nor the influence on precursors that reduce initiation of sex. We used data from a longitudinal study of AI/AN youth attending all middle schools from a Northern Plains reservation from 2006–2009 to estimate a structural equation model based on a cultural and age adapted theoretical framework. The tested model included frequent residential mobility as the independent variable and sex refusal self-efficacy as the dependent variable. Mediating variables included factors related to individual risks, psychological well-being, and social supports. Results indicate a direct association between residential mobility and sex refusal self-efficacy (-.29, p = 0.05) and an indirect association mediated by deviant peers (-.08, p = .05). Other mediating variables did not provide insight on the mechanism by which residential mobility influences skills to refuse sexual intercourse among AI/AN youth in early adolescence. Findings provide evidence for an association between residential mobility and precursors to sexual experience suggesting augmenting sexual health interventions for highly mobile youth.

## Introduction

Frequent residential mobility and risky sexual behavior among adolescents are linked; youth who experience multiple lifetime moves are more likely to report initiation of sex at early ages [1–3]. Adolescent pregnancy has also been shown to be positively correlated with the number of residential moves experienced early in life [4, 5]. This link may help explain disproportionate rates of sexual risk among American Indian (AI) youth. AIs report residential mobility at a rate nearly four times that of non-Hispanic Whites [6–8], and AI youth ages 15–19 experience higher levels of risky sexual activity and subsequently, elevated rates of sexually transmitted diseases and teen pregnancy compared to their non-Hispanic White peers [9, 10]. But while these statistics exist in tandem and suggest a link, a direct association has not yet been investigated in this population.

Understanding the mechanisms through which residential mobility might impact sexual risk among AI youth could inform effective intervention design and help reduce health disparities. True prevention, however, requires intervening prior to onset of sexual risk behavior, in this case with young adolescents. Research to date on the links between residential mobility and sexual risk has focused on older youth and on behavioral outcomes, such as sexual activity and pregnancy, that are premature for young adolescents. Rates of these behaviors in the pre-teen and early teen years are low, and once they occur it may be too late for prevention efforts. Informing prevention will depend on identifying and measuring the precursors to these behaviors and understanding how precursors are related to risk factors, such as residential mobility. In this study, we built on prior theoretical and empirical work among older teens but focused on sex refusal self-efficacy [1], a well-established precursor of sexual risk among AI youth. We used structural equation modeling (SEM) to examine how residential mobility was related to this early indicator of sexual risk among AI young adolescents of a Northern Plains tribe [11].

### American Indian mobility and youth sexual risk-taking

AIs have a long history of residential mobility. Beginning in the 1800s, federal policies and broken treaties led to forced migration of eastern tribes to the west [6, 12]. Forced local and regional residential concentration was also common, as AI communities were pushed to increasingly shrinking tracts of tribal lands[13]. From the 1950s into the 1970s, many AIs moved from tribal reservations to urban areas under the Bureau of Indian Affairs Direct Relocation Program—a policy that reduced U.S. government economic support to reservations by assimilating AIs into the urban workforce [14]. While the policy officially ended with the Self-Determination and Educational Assistance Act of 1975, multiple moves over a life time continue for many AIs [15]. These moves are often in response to adverse social and economic conditions disproportionately common among AIs [16–18] and are known determinants of mobility among the general population [8].

Sexual risk for AI youth unfolds within this historical context of AI mobility; residential mobility may be an important risk factor exacerbating the elevated rates of risky sexual behaviors and subsequent adverse sexual health outcomes experienced by AI adolescents. In other populations, frequent residential mobility has been shown to increase the likelihood of early sexual experience, multiple sexual partners, and teen pregnancy[1]. Literature has found that mobility combined with adverse social factors, such as poverty, exacerbates sexual risk taking among adolescents [19]. This is particularly disconcerting considering the elevated socioeconomic disadvantages experienced by many tribal communities[18]. Moreover, the significance of investigating the influence of frequent residential moves on precursors to sexual risk taking among AI young adolescents becomes evident when considering the clear disparities in sexual health outcomes that originate in early adolescence. For example, AI youth who initiate sexual activity at young ages are three times less likely to use a condom and two times more likely to report having more than one sexual partner compared to those who initiate later [20, 21]. These behaviors place AI youth at greater risk for sexually transmitted infections (STIs) and for pregnancy [22, 23]. Assessing the ways in which residential mobility may operate in early adolescence to increase sexual risk may provide an opportunity to decrease disparities in later teen years.

#### Theoretical background

This study was guided by a conceptual model developed by South et al.[1], who described the effects of residential mobility on premarital intercourse on youth in grades 7–11 [1]. Their model categorized theories describing how the effect of residential mobility influences adolescent sexual experience into four “explanatory rubrics”—individual risk behaviors, parent-child relationships, psychological well-being, and peer social networks [1]. The authors theorized that youth who move frequently have lower grade point averages (GPAs) and participate in fewer extracurricular activities than their non-mobile classmates. Diminished academic performance and limited engagement in school activities–individual risks–are associated with early sexual activity. They also posited that strong connections between children and parents encourage normative guidance that facilitates positive development. Residential mobility may be potentially problematic for adolescents because parent-child relationships are disrupted as parents may be preoccupied with resettlement and lessen their engagement and involvement in their child’s life. In turn, weak parent-child relationships increase sexual activity. The authors identified psychological well-being as a mediator as adolescence is considered a time of substantial turmoil due to factors associated with puberty and the struggle with self-identity. The additional stress of residential mobility enhances this sense of uncertainty resulting in the development of depression or anxiety. Adolescents, subsequently, attempt to ameliorate this psychological distress by engaging in sexual intercourse. Finally, South and colleagues hypothesized that peer networks play a paramount role in shaping adolescent behaviors. In their model, residential mobility affects adolescent sexual risk taking because newcomers are more likely to be integrated and accepted into peer groups whose members encourage deviant, risky, or problematic behaviors. As a result, mobile youth experience increased exposure to peers whose values are conducive to early and risky sexual intercourse.

South et al. evaluated these potential mediators between the relationship of residential mobility and sexual initiation using a sample of youth from the US population. A formal mediational analysis was not conducted; instead the authors conducted successive multivariate logistic regression models incorporating potential mediators to examine their ability to account for residential mobility on timing of first intercourse. Only peer networks were shown to be a significant factor; deviant peer networks were related to adolescent sexual experience among frequent movers.

Despite the initial lack of empirical support, the theoretical basis of South’s model provided a useful heuristic framework for our investigation of the relationship between residential mobility and sexual risk among AI youth. We believed the potential mechanisms of the effect of mobility could be better understood with formal mediation analyses. We were also specifically interested in how they operated among AI youth. Thus, we included parent-child relationships, psychological well-being, and peer group affiliation as key mediators between residential mobility and a precursor to sexual risk taking in our exploratory model. We hypothesized that residential mobility would put youth at sexual risk by disrupting the protective relationship between parents/guardian and children, increasing psychological distress, and increasing the likelihood youth will assimilate to behaviors ascribed by deviant peers [1]. In the following, we describe the theoretical basis for the hypothesized relationships of our model.

#### Final exploratory model

**Fig 1:**
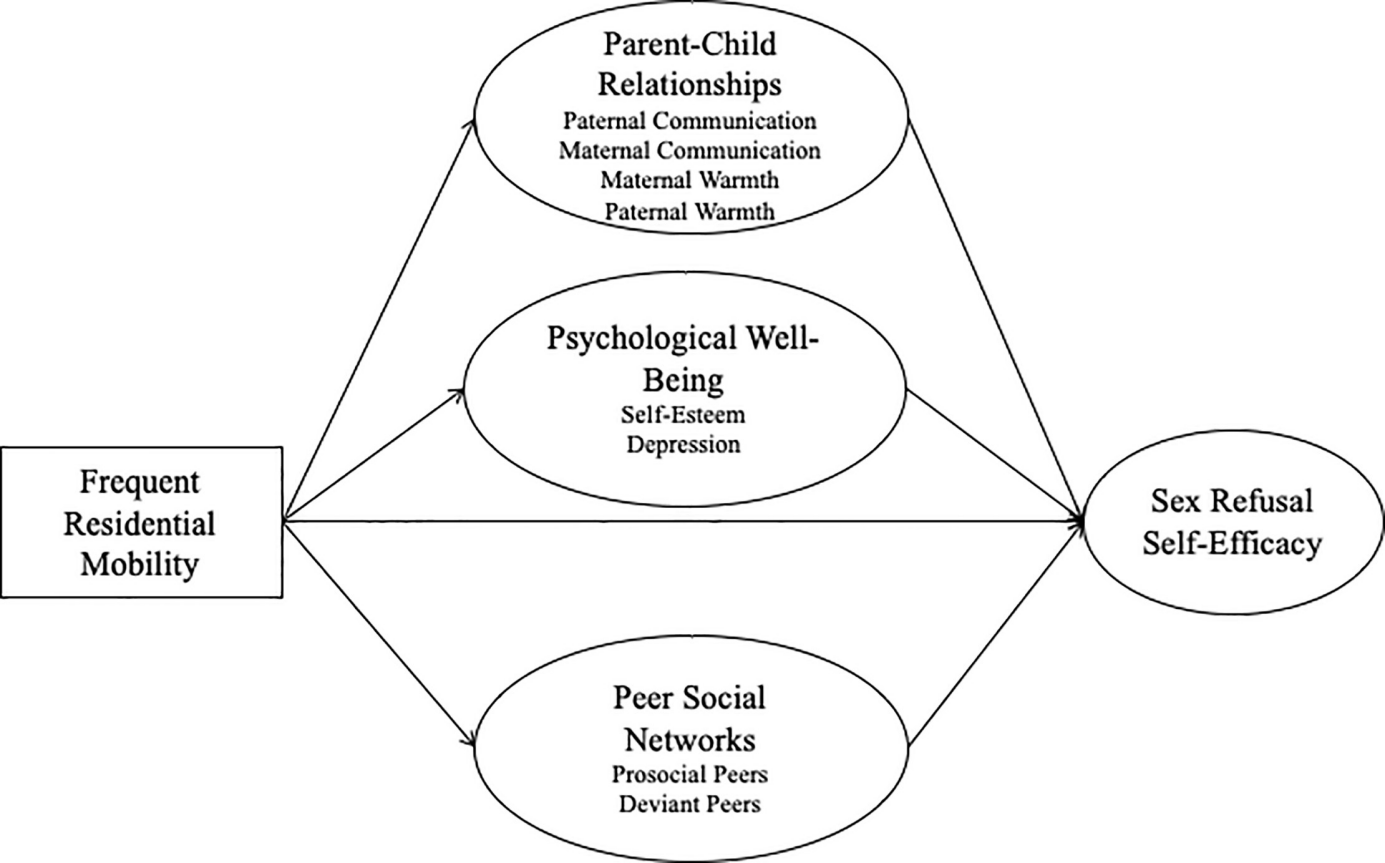
Proposed exploratory model of the direct effect of residential mobility on sex refusal self-efficacy and the plausible mediators. Direct effect between residential mobility and sex refusal-self efficacy and potential mediators of qualities of parent-child communication, psychological well-being, and peer social networks.

Sex refusal self-efficacy and residential mobility. We adapted the model for the developmental stage of our youth, namely early adolescence (Fig 1). In this developmental stage, sexual experience is less salient; thus, we focused on a precursor to sexual behavior. We considered several measures of psychosocial precursors and chose sex-refusal self-efficacy as the most relevant since this construct has been shown to predict subsequent sexual experience in AI youth [24]. Sex refusal self-efficacy is the ability of youth to resist social pressures to engage in risky sexual behavior. Early adolescence begins the stage in which youth socially engage with their peers independently without parental or guardian supervision [25]. This increases the likelihood that youth will encounter social pressures to participate in risky behaviors that they may be unable to evade [26]. The stronger an adolescent’s perceived sex refusal self-efficacy the less likely they are to engage in early or risky sexual behaviors, despite pressure from peers to do so [11]. Moreover, while literature on the direct relationship between residential mobility and sex refusal self-efficacy does not exist, researchers have found that residential mobility is often associated with reduced competence [1, 27], a broader construct conceptually similar to self-efficacy. Thus, we hypothesized that residential mobility will be negatively associated sex refusal self-efficacy.

Sex refusal self-efficacy and parent-child relationships. Encouragement by parents that an adolescent has the ability to achieve his or her goals has been shown to be a source of self-efficacy during childhood [28]. An increase in positive parent-child interactions is associated with an increase in child self-efficacy [29]. Research about parental influence on adolescent self-efficacy is thin. However, ample research demonstrated that positive parent-child relationships can help delay adolescent sexual debut and reduce risky sexual behaviors [30–33], perhaps by increasing adolescents’ sex refusal self-efficacy which in turn delays the onset of sexual experience. Thus, we hypothesized that parent-child relationships would be positively associated with sex refusal self-efficacy among AI young adolescents.

Psychological well-being and sex refusal self-efficacy. An individual’s psychological and emotional state is a crucial factor in the construction of self-efficacy during childhood and adolescence. We found no research on the relationship between psychosocial factors and sex-refusal self-efficacy, specifically. However, researchers have found that general self-efficacy, physical self-efficacy, and academic self-efficacy are significantly negatively related to depression and anxiety [34]. Therefore, we hypothesized that psychological well-being, depression and self-esteem, would be negatively associated with sex refusal self-efficacy among AI young adolescents.

Peer Social Networks and sex refusal self-efficacy. During adolescence, peers assume some of the socialization functions formerly carried out by parents and adult guardians [27]. Unfortunately, research on the extent that self-efficacy is influenced by peers is not definitive. Some researchers argue that self-efficacy is strongly influenced by peers, while others argue that it is influenced by personal success, failure, and affect [27, 35–37]. However, researchers have consistently found that adolescents who have competent peers eventually become equally competent [27], and, as previously mentioned, competency is a construct related to self-efficacy. Researchers attribute this to the adolescent’s learning the knowledge and skills necessary to accomplish the task by observing competent peers [27]. Therefore, we hypothesized that sex refusal self-efficacy would be positively associated with prosocial peer group affiliation and negatively associated with deviant peer group affiliation.

Within our sample there was little variance in individual risk behaviors related to school, the mean GPA was nearly 3.0 and over 90% participated in at least one extracurricular activity, therefore we omitted individual risk behaviors.

## Methods

### Study sample

Data were from the Wiconi Teca Waste Study (Wiconi), a longitudinal clustered-randomized trial conducted on a Northern Plains reservation, one of the poorest regions in the United States [38], from 2006–2009. The purpose of Wiconi was to assess the effectiveness of a theory-based HIV preventive intervention, Circle of Life, among AI youth. All 13 middle schools across the reservation participated. Eligibility was based on youth enrollment in middle school and assent and parent/guardian consent for participation; study details are described elsewhere [38]. The study was approved by the Colorado Multiple Institutional Review Board and by the Tribal Research Review Board. For this analysis, only baseline waves of data from participants in the wait-listed groups (N = 327) were used.

### Survey measures

The Wiconi study used measures from the National Longitudinal Study of Adolescent Health; the American Indian Service Utilization, Psychiatric Epidemiology, Risk and Protective Factors Project (AI-SUPERPFP) Instrument; the Panel Study of Income Dynamics: Child Development Supplement of the Family Economics Study (PSID); and the Rosenberg Self-esteem inventory [39–42]. Measures utilized here have been validated for AI youth [38, 43]. All measures used were reviewed by community focus groups, field staff, and investigators for developmental and cultural appropriateness. The final factors included in our analyses were identified through Exploratory Factor Analysis (EFA) using Varimax orthogonal rotation and maximum likelihood estimation with SPSS v.24. We examined the communalities, explained variance, factor loadings, and scree plots to identify the final factors used in this analysis. We then examined the internal consistency of each factor using Cronbach’s alpha.

#### Residential mobility

Youth were asked, “Not counting the place where you live now, how many other houses or places did you live in during the past 12 months?” Youth were classified as non-frequent movers if they reported living in a different house or place 0–1 times in the past 12 months and were classified as frequent movers if they moved 2 or more times in the past 12 months. The frequency of responses to this item supported dichotomization of the variable allowing for viable analysis; most participants who reported moving more than once within a year moved two times. Moreover, a dichotomous variable is consistent with the measure used in the South et al. study, and thus we were able to maintain comparability in our approach.

#### Sex refusal self-efficacy

EFA identified a single factor for sex-refusal self-efficacy consisting of the following four items (α = .75). Youth were asked how likely they were to say no to each of the following: 1) “having sex with someone they wanted to go out with again,” 2) “having sex with someone if neither partner had a condom,” 3) “having sex with someone they already had sex with,” and 4) “having sex after they have been drinking alcohol.” Responses were recorded on a four-point Likert scale (4 = I’d definitely say “no”, 3 = I’d probably say “no”, 2 = I’d probably say “yes”, and 1 = I’d definitely say “yes”). To note, we acknowledge that in some communities sex refusal self-efficacy may not be culturally valid. That is, asking to use a condom may be interpreted as a sign of distrust and practicing sex without a condom is a sign of love, and thus youth may report lower sex refusal self-efficacy [44, 45]. However, focus groups were conducted in the AI community from which these data come from on culture, context, and sexual risk taking among adolescents. This study found that adolescents understood the importance of condom use and youth did not mention infidelity or disrespect with use of condoms. In fact, condom use was linked with self-respect and dignity—characteristics that are associated with AI cultural tradition and pride[46].

#### Parent-child relationship quality

Parent-child relationship quality consisted of four factors identified through EFA: 1) maternal communication (α = .83), 2) paternal communication (α = .83), 3) maternal warmth (α = .82), and 4) paternal warmth (α = .87). The following are the items included in the communication factors, youth were asked how often they talked to their mother/father (female/male guardian) about how things are with friends, plans for future, and problems at school. The warmth factors included items that asked youth how often their mother/father enjoys doing things with them, cheered them up when they were sad, praised them, and gave them a lot of care and attention. Youth responded to all items on a five-point Likert scale (1 = never, 2 = once or twice, 3 = about once a week, 4 = 2–3 days a week, 5 = almost every day or every day).

#### Psychological well-being

Self-esteem was measured using six items from Rosenberg[47], rated on a four-point Likert scale (1 = Strongly disagree, 2 = Somewhat disagree, 3 = Somewhat agree, 4 = Strongly agree). EFA identified two factors of self-esteem, negative self-esteem (α = .76) and positive self-esteem (α = .71). Negative self-esteem items included: I feel worthless, I think I am not good at all, and I feel that I am a failure. The positive self-esteem items included: I feel that I may have many good qualities, I have a positive attitude, and I am satisfied with myself.

Depression (α = .88) was measured using items from the University of Michigan Clinical Interview for Depression (UM-CID) modified for AI use; items were adapted to address concerns of overly complex language brought up by community informants [42]. The seven items asked youth how often they felt they could not stop feeling down or sad, depressed, like a failure, fearful, lonely, and had crying spells in the past week. Responses range from 1 = rarely/none of the time to 4 = most or all of the time.

#### Peer social networks

Two factors for peer social networks were identified through EFA and included: 1) prosocial peers (α = .63) and 2) deviant peers (α = .70). Specific to prosocial peers, participants were asked how many of their friends: were likely to volunteer or participate in community groups; go to Inipi (sweat/ceremony) or church regularly; think schoolwork is very important; and plan to go to college. Related to deviant peers, youth were also asked how many of their friends encourage disobeying parents, try to get you to do dangerous things, get in a lot of fights with other kids, and get in trouble at school. Responses to all items were recorded as 1 = None, 2 = A Few, 3 = Some, 4 = Many, and 5 = Almost all or all.

### Analyses

SPSS v. 24 was used for sample descriptives [48]. Mplus v. 7.3 was used to conduct confirmatory factor analysis (CFA) and structural equation modeling (SEM)[49]. We chose SEM for our analysis because this method allows for a formal mediational model to analyze complex constructs of multiple manifest variables—as opposed to a single observed variable [50]. Further, unlike conventional path analysis, SEM incorporates measurement error when analyzing the mediational relationships, thus reducing bias in parameter estimates [50]. Estimation biases related to missing data are also reduced with this approach. First, the conventional use of regression modeling combines the separate results of two or more equations, which can be problematic when missing data varies in the different equations [50]. Second, the use of Mplus for model estimation has the advantage of using full information maximum likelihood (FIML) estimation. FIML has been shown to provide unbiased and efficient estimates for missing data [51], thus overcoming fundamental issues with data missingness often encountered in other methods for estimation of mediational relationships.

To conduct our analysis, we began with CFA to validate the measures included in our model. Three primary fit indices were used to examine model fit: the comparative fit index (CFI), the root mean square of approximation (RMSEA), and the standardized root mean square residual (SRMR). Model fit was considered acceptable if the CFI was greater than .90, the RMSEA was less than or equal to .05, and the SRMR less than or equal to .08. In addition, residuals and R-squares were examined [50]. SEM was then employed to build our statistical model.

After confirming our measurement model, considering this model is exploratory in nature, we felt that it was best to employ a stepwise approach to specifically identify the statistically significant mediational relationships to include in our final model. We first looked at the direct relationship each individual mediator had with sex refusal self-efficacy and residential mobility, separately. We then looked at the indirect relationships, that is, the relationships between residential mobility and sex refusal self-efficacy through each individual mediator. The mediators that showed significant direct and indirect relationships were included in our final model. We decided not to analyze the full exploratory model or a saturated model because these approaches would limit our ability to identify the specific significant direct and indirect relationships of our exploratory model. To formally test for mediation, we used the MODEL INDIRECT function which computed both direct and indirect standardized path coefficients, errors, and calculated probabilities [47]. We originally analyzed the data using a weighted least square mean and variance adjusted estimator (Estimator = WLSMV) for these analyses. We then ran our models using a maximum likelihood robust estimator with Huber-White covariance adjustment (Estimator = MLR) to address data missing at random (MAR). There were slight differences in the results of the analyses between both estimators. Because the WLSMV estimator cannot handle data MAR given its pairwise variable orientation we decided to use the results of the analyses using the MLR estimator [52]. All analyses adjusted for the clustered sampling strategy of the Wiconi study, gender, and GPA. The same indices used for CFA were used to assess model fit of our SEM.

## Results

### Sample descriptive statistics

Table 1 presents descriptive statistics of the sample by residential mobility status. Nearly one-quarter were classified as frequent movers. Among non-movers, the sample was nearly evenly split between boys and girls; in contrast, two-thirds of movers were girls. Movers were significantly more likely to report receiving food stamps and commodities—a federal food assistance program offered to participating American Indian Tribal Organizations. As shown in Table 1, no differences between frequent and non-frequent movers were found in sex refusal self-efficacy or any of the proposed mediating variables except paternal warmth. Adolescents who moved less frequently had a higher mean paternal warmth compared to frequent residential movers.

**Table 1:** Descriptive statistics of the Wiconi sample characteristics and study variables by residential mobility status.

Variable	Non-frequent mover (n = 252, 77.1%)	Frequent Movers (n = 75, 22.9%)	
Sample characteristics	M (SD)	M (SD)	P-value
Female	50.4%	66.7%	.02
Age	12.96(.74)	12.97(.74)	.86
GPA	3.00(.66)	2.90(.67)	.08
Extracurricular activities (yes)	91.0%	94.7%	.64
Family receives food stamps	46.9%	67.1%	.03
Family receives food commodities	34.2%	49.3%	.03
Dependent variable			
Sex refusal self-efficacy* (scale 1–4, alpha .75)	1.51(.62)	1.59(.61)	.38
Mediating variables			
Maternal Communication* (scale 1–3, alpha = .83)	3.14 (1.28)	3.12(1.26)	.46
Paternal Communication* (scale 1–3, alpha = .83)	2.96(1.27)	2.93(.50)	.48
Maternal warmth* (scale 1–4, alpha = .82)	2.43(.50)	2.37(.51)	.35
Paternal warmth* (scale 1–4, alpha = .87)	2.44(.53)	2.25(.66)	.03
Negative self-esteem* (scale 1–4, alpha = .76)	3.59(.64)	3.43(.63)	.07
Positive self-esteem* (scale 1–4, alpha = .71)	3.12(.72)	3.01(.69)	.32
Depression* (scale 1–7, alpha = .88)	3.11(.71)	3.02(.70)	.35
Prosocial Peers* (scale 1–4, alpha = .63)	2.97(.86)	3.00(.86)	.80
Deviant Peers* (scale 1–4, alpha = .70)	1.98(.77)	2.09(.68)	.23

*note the direction of scale scores–higher scores more sex refusal self-efficacy, deviant peers, etc.

### Structural equation modeling

CFA identified good model fit for most of our measurement models in our analyses, as illustrated in Table 2. The RMSEA for the models mediated by deviant and prosocial peers indicated mediocre; MacCallum et al. suggests that a RMSEA of .08 is considered a mediocre, yet acceptable fit [53]. Moreover, the CFI and SRMR for both peer social network models suggested a good fit.

**Table 2:** Confirmatory factor analysis of the direct and indirect relationships of the exploratory model.

Measurement Model			
Direct	CFI*	RMSEA**	SRMR***
Residential mobility to sex refusal self-efficacy	.97	.05	.04
Indirect			
Through maternal communication	.91	.05	.06
Through paternal communication	.96	.05	.05
Through maternal warmth	.91	.05	.05
Through paternal warmth	.90	.05	.05
Through depression	.96	.05	.04
Through positive self esteem	.91	.05	.05
Through negative self esteem	.92	.05	.05
Through prosocial peers	.97	.06	.04
Through deviant peers	.98	.07	.04

*CFI = comparative fit index

**RMSEA = root mean square error of approximation

***SRMR = standardized root mean square residual

**Fig 2:**
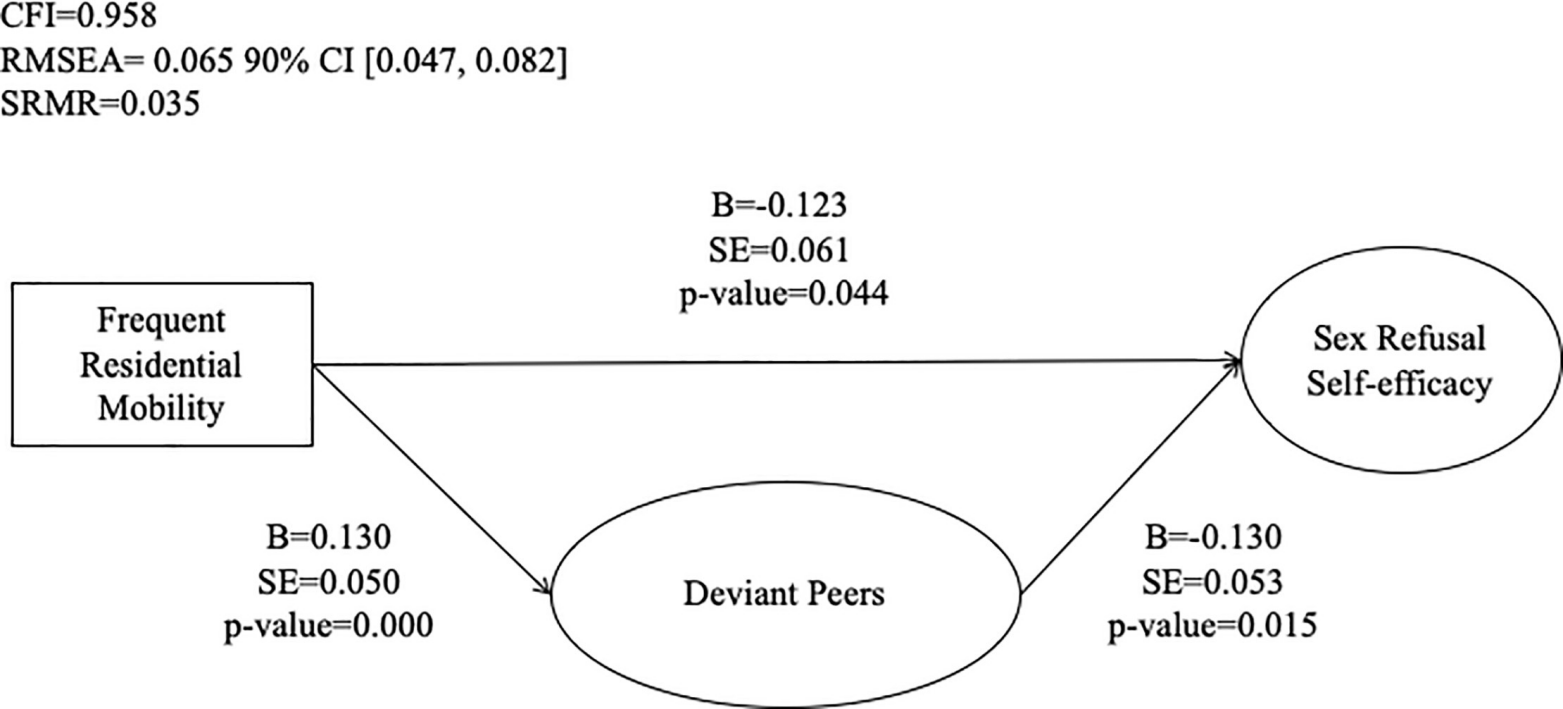
Significant direct and indirect relationships between residential mobility and sex refusal self-efficacy. There was a significant direct effect between residential mobility and sex refusal self-efficacy. The only indirect effect among the dependent and independent variable was through deviant peer group affiliation.

Findings from our structural equation analyses are presented in Fig 2 and Table 3. Model fit indices for all direct and indirect structural models were reviewed, the indirect models that included positive self-esteem and deviant peers were considered an excellent fit according to their CFI and SRMR, however, RMSEA suggested mediocre fit. All other mediational models were an excellent fit across all model fit criteria. A significant direct effect of residential mobility on sex refusal self-efficacy was observed. Other significant direct effects included maternal warmth, paternal warmth, positive self-esteem, prosocial peers, and deviant peers on sex refusal self-efficacy. The effects of paternal and maternal warmth, positive self-esteem and prosocial peers on sex refusal self-efficacy were positive. In addition, there was a negative direct effect of residential mobility on maternal communication and a positive direct effect on negative self-esteem and deviant peers. The only significant indirect effect between residential mobility and sex refusal self-efficacy was through deviant peers (Fig 2).

**Table 3:** Coefficients for structural equation mediational models of the direct and indirect relationships between residential mobility and sex refusal self-efficacy.

Structural Relationships				
Residential mobility to sex refusal self-efficacy	**B**	**se**	**p**	**β**
Direct effect	-.29	.15	.05*	-.12
Model fit	CFI = .97, RMSEA = .04, SRMR = .03			
Through maternal communication				
Residential mobility to maternal communication	-.26	.12	.03*	-.10
Maternal communication to sex refusal self-efficacy	.05	.07	.52	.07
Indirect effect	-.01	.02	.60	-.01
Model fit	CFI = .97, RMSEA = .05, SRMR = .04			
Through paternal communication				
Residential mobility to paternal communication	-.19	.28	.50	-.11
Paternal communication to sex refusal self-efficacy	-.03	.04	.54	-.04
Indirect effect	.01	.01	.72	.00
Model fit	CFI = .98, RMSEA = .03, SRMR = .04			
Through maternal warmth				
Residential mobility to maternal warmth	-.04	.12	.09	-.02
Maternal warmth to sex refusal self-efficacy	.20	.08	.01*	.18
Indirect effect	-.01	.03	.72	.00
Model fit	CFI = .99, RMSEA = .05, SRMR = .02			
Through paternal warmth				
Residential mobility to paternal warmth	-.02	.20	.94	-.01
Paternal warmth to sex refusal self-efficacy	.12	.06	.05*	.14
Indirect effect	.00	.02	.94	.00
Model fit	CFI = .98, RMSEA = .05, SRMR = .03			
Through depression				
Residential mobility to depression	.11	.11	.32	.07
Depression to sex refusal self-efficacy	.16	.13	.22	.23
Indirect effect	.02	.03	.48	.01
Model fit	CFI = .92, RMSEA = .00, SRMR = .04			
Through positive self esteem				
Residential mobility to positive self esteem	-.22	.12	.08	-.09
Positive self-esteem to sex refusal self-efficacy	.38	.09	.00*	.28
Indirect effect	-.08	.06	.17	-.04
Model fit	CFI = .96, RMSEA = .06, SRMR = .03			
Through negative self esteem				
Residential mobility to negative self esteem	.24	.11	.03*	.14
Negative self-esteem to sex refusal self-efficacy	-.11	.07	.15	-.08
Indirect effect	-.03	.03	.33	-.01
Model fit	CFI = .97, RMSEA = .05, SRMR = .03			
Through prosocial peers				
Residential mobility to prosocial peers	.03	.07	.69	.02
Prosocial peers to sex refusal self-efficacy	.37	.11	.00*	.22
Indirect effect	.01	.02	.68	.00
Model fit	CFI = .97, RMSEA = .05, SRMR = .03			
Through deviant peers				
Residential mobility to deviant peers	.24	.10	.01*	.13
Deviant peers to sex refusal self-efficacy	-.32	.08	.00*	-.25
Indirect effect	-.08	.04	.05*	-.03
Model fit	CFI = .96, RMSEA = .08, SRMR = .04			

*CFI = comparative fit index, RMSEA = root mean square error of approximation, SRMR = standardized root mean square residual

*P-value less than or equal to .05 indicates a significant relationship

## Discussion

AI families change residences more often than do non-Hispanic White families. We theorized that mobility likely has important ramifications for sexual risk taking that would be evidenced in early adolescence as lower sex refusal self-efficacy. To our knowledge, ours is the first study to systematically investigate this relationship. Through the use of SEM, our study sought to understand the complex direct and indirect relationships that might account for the positive relationship between residential mobility and teen sexual experience. Our findings were similar to those of South et al.[1], finding an indirect relationship, mediated by peer group affiliation, between residential mobility and sexual risk taking. Our replication of South et al.’s study with a precursor of sexual risk in a younger sample and in a population at high risk due to high mobility has important implications for preventive intervention. Specifically, our study identified two potential points of sexual risk intervention for AI youth—multiple moves and deviants peers. For instance, establishing programs that intentionally connect students who are new to a school with peers who have high academic performance and are involved in school activities may help positively transition frequent movers.

Our study did not identify significant indirect relationships between residential mobility and sex refusal self-efficacy mediated by parent-child relationships and psychosocial well-being factors. However, these findings are indeed still informative and provide insight to future research efforts to better understand the mechanism by which residential mobility influences the development of sex refusal self-efficacy among AI adolescents. Further, we have identified specific adaptations to our exploratory model to consider for future analysis. First, perhaps the items used to construct parental communication do not influence sex-refusal self-efficacy. This study measured general parental communication; communication about sex and sexuality may be more relevant to sex refusal self-efficacy for AI adolescents. For instance, researchers have found that children of parents who discussed how to delay sex and sex refusal were less likely to report sex during adolescence. Parent-child sexual risk communication has been shown to associated with greater self-efficacy related to sex and sexual communication with partners.[54] Lack of parent-child communication around sex has been shown to cause adolescents to turn to peers that may influence their sexual behaviors.[55] In addition, in many AI communities uncles and aunts impart knowledge on values and provide guidance around character building and life skills [56, 57]. Cultural adaptation of the South et al. model may be necessary to integrate the greater reliance on extended family with the development of risk prevention skills. By focusing solely on parent-child relationships we may have limited our ability to understand the mechanism by which residential mobility influences sex refusal self-efficacy. That is, as youth move frequently relationships among their extended family may weaken or are lost; in result, the skills to refuse sex may not be learned. Also related to the need of cultural adaptation, a body of literature documents notable resilience in the face of oppression, hostility, and other forms of adversity among AIs [58]. Perhaps, residential mobility may not have influenced the psychological well-being because AI youth have developed skills to more easily cope and adapt when facing stress. Thus, it may be useful to integrate protective factors, such as resilience, into a future exploratory model to better conceptualize our understanding of the mechanism by which residential mobility influence sex refusal self-efficacy.

Our analyses are subject to several notable limitations. First, the study used only cross-sectional baseline data from a larger longitudinal study, precluding causal interpretations. Longitudinal analyses monitoring moves and adolescent development prospectively will provide important insights to the findings presented here. We also did not distinguish between short and long-distance moves. Future studies may benefit from examination of these distinctions, since long-distance moves may mean a greater loss of important social connections and create greater barriers to successful transitions to new settings. Second, self-report of sex refusal self-efficacy is an approximate measure of risky sexual behaviors. However, the prevalence of sexual experience and risky sexual behaviors among young adolescents is low and thus sexual behavior outcomes such as vaginal intercourse or sexual initiation may not be appropriate. Identifying more age-appropriate outcomes for this particular age group was critical for developmentally relevant analyses. Further, sex refusal self-efficacy has been shown to be a particularly viable predictor of subsequent sexual behaviors among AI youth from the community in which these data come from [11]. Also, these analyses assumed that the relationships among residential mobility and the mediating variables were the same across genders; sample size limited our ability to test gender-specific relationships. Last, these data were collected in one tribal community and the results reported here may not be generalizable to other AI youth. Yet despite these limitations, these findings are informative for the development of sexual-risk reduction efforts among AI young adolescents: those experiencing frequent residential mobility are at-risk, and an important pathway of risk is through the influence of deviant peers.
